# Deciphering the First Mitochondrial Genome of the *Liolophura* Pilsbry, 1893 Genus: An Extensive Phylogenetic Study Within the Chitonidae Family

**DOI:** 10.3390/genes16050606

**Published:** 2025-05-20

**Authors:** Qianqian Zhou, Zhiyong Liu, Weifeng Dong, Bingpeng Xing, Site Luo, Peng Xiang

**Affiliations:** 1 School of Life Sciences and Technology, Tongji University, Shanghai 200092, China; 2 Third Institute of Oceanography Ministry of Natural Resources, Xiamen 361005, China; 3 Xiamen Ocean Center Ministry of Natural Resources, Xiamen 361008, China; 4 Schmid College of Science and Technology, Chapman University, Orange, CA 92866, USA; 5 School of Life Sciences, Xiamen University, Xiamen 361102, China

**Keywords:** *L. japonica*, gene arrangement, chitonidae evolution, polyplacophora

## Abstract

**Background**: The Polyplacophora class, which includes all chitons, is distinguished by its unique eight-piece interlocking armor, showcasing a vast diversity in marine environments. However, the detailed evolutionary relationships within the Chitonidae family remain largely unknown. The mitochondrial genome is essential for understanding these relationships, but there has been a significant lack of such genomic information, especially for the *Liolophura* genus. **Methods**: We generated the first mitogenome of *Liolophura japonica* by assembling Illumina reads with GetOrganelle, polishing with Pilon, annotating genes with MitoZ and MITOS2, and inferring phylogeny from 13 concatenated protein-coding genes (PCGs) using MAFFT and IQ-TREE. **Results**: The mitogenome is 15,209 base pairs long and includes 13 protein-coding genes, 22 transfer RNAs, and 2 ribosomal RNAs. The mitogenome exhibited a slight AT bias common in Chitonidae and showcased structural uniqueness with no control region found. Notably, all protein-coding genes demonstrated evidence of purifying selection, with Ka/Ks ratios below 1, highlighting evolutionary conservation. Phylogenetic analysis reveals a close relationship between *L. japonica*, *Acanthopleura loochooana* Broderip & Sowerby 1829, and *Acanthopleura vaillantii* Rochebrune, 1882, potentially warranting future taxonomic re-evaluation. This research emphasizes the crucial role of mitochondrial genomes in mollusk phylogeny and sets the stage for advanced genetic studies within this group. **Conclusions**: The significance of this study lies in its contribution to understanding the mitochondrial genome of *L. japonica*, a key species within the Polyplacophora class. By analyzing its mitogenome, we aim to enhance our understanding of evolutionary processes in chitons and other mollusks.

## 1. Introduction

Mitochondria, colloquially known as the cell’s powerhouses, play pivotal roles beyond energy metabolism, including in apoptosis, aging, and the pathology of various diseases [1]. The mitochondrial genome (mitogenome), typically existing as a circular double-stranded DNA molecule, is widely used in systematic and evolutionary studies due to its compact gene structure, high copy number, maternal inheritance, elevated mutation rate, and ease of extraction [2]. These characteristics make mitochondrial DNA an ideal molecular marker for investigating recent evolutionary events, genetic diversity, and lineage differentiation, especially at the species and population levels [3]. It has proven particularly powerful in species identification, phylogeography, and in revealing fine-scale evolutionary relationships. Although its rapid evolutionary rate and substitution saturation may limit its effectiveness in resolving deeper phylogenetic nodes, mitogenomes remain a valuable resource for reconstructing phylogenies among closely related taxa and for tracking gene flow, demographic history, and biogeographic patterns [4].

The class Polyplacophora, commonly known as chitons, is characterized by eight articulating shell plates that provide both protection and flexibility—an arrangement shared across all families within the group. Chitons are widely distributed from shallow intertidal zones to deep-sea habitats and possess a specialized radula, a rasping tongue-like organ typical of most mollusks except bivalves, which enables them to graze effectively on algal biofilms. The genus *Liolophura* Pilsbry, 1893, belonging to the family Chitonidae, retains these ancestral traits but also shows population-level variation in shell coloration, body size, and habitat preference [5,6,7]. *L. japonica*, commonly called the Japanese chiton, is distributed across the northwestern Pacific, particularly along the coasts of China, South Korea, and Japan. This species occupies rocky intertidal zones and primarily feeds on microalgae, consistent with its generalist herbivorous lifestyle.

Previous studies have uncovered three distinct genetic lineages within *L. japonica*, suggesting potential cryptic speciation within what was considered a single species [7]. These findings highlight the taxonomic complexity within the genus and underscore the need for more comprehensive molecular datasets. Although a single mitogenome cannot resolve lineage boundaries or confirm cryptic species status, it provides foundational data on gene order, structural features, and selective constraints. Here, we report the complete mitochondrial genome of *L. japonica*, which contributes to expanding genomic resources for the genus and facilitates future comparative studies aimed at clarifying its phylogenetic relationships within Polyplacophora.

This study aims to report and characterize the complete mitochondrial genome of *L. japonica*, assembled and annotated using next-generation sequencing techniques. While only a single representative of Chitonidae is included, the resulting mitogenome provides essential baseline information on gene content, structure, and compositional patterns. This genomic resource may serve as a reference for future comparative studies and facilitate broader phylogenetic investigations within Polyplacophora, especially when additional taxa are sequenced.

## 2. Materials and Methods

### 2.1. Ethical Considerations

All procedures involving animal samples in this study adhered strictly to ethical guidelines and regulations for the care and use of laboratory animals. The Ethics Committee of the Third Institute of Oceanography Ministry of Natural Resources approved the study protocol. Protocol No. TIO189935 dated 10 May 2019.

### 2.2. Specimen Collection and DNA Extraction

Specimens were randomly collected by hand from intertidal rocky shores in Xiamen, China (24°27′ N, 118°04′ E), during low tide in June 2019. After morphological examination, five adult individuals were identified as *L. japonica* based on diagnostic characteristics, and one individual was randomly selected for genomic DNA extraction. All specimens were transported to the laboratory in seawater-filled containers to maintain physiological conditions. Species identification was confirmed by Professor Peng Xiang using established morphological criteria. Genomic DNA was extracted from the adductor muscle tissue of the selected specimen using the TIANamp Marine Animal DNA Kit (Tiangen Biotech, Beijing, China), following the manufacturer’s protocol. DNA quality and concentration were assessed using agarose gel electrophoresis and a NanoDrop 2000 spectrophotometer (Thermo Fisher Scientific, Waltham, MA, USA).

### 2.3. Library Preparation and Sequencing

Approximately 1 μg of high-quality genomic DNA was used to prepare the sequencing library. The library was constructed with an average insert size of 350 bp, using the TruSeq DNA Sample Prep Kit (Illumina, San Diego, CA, USA) according to the manufacturer’s protocols. Sequencing was performed on an Illumina NovaSeq 6000 platform by Novogene Corporation (Beijing, China), generating paired-end reads of 150 bp in length.

### 2.4. Genome Assembly and Annotation

The mitogenome was assembled from the sequencing data using the GetOrganelle v1.7.7.1 pipeline, with default parameters to ensure the accuracy of the assembly [8]. The ‘animal_mt’ database provided the seed reads for initiating the assembly process. BWA v0.7.19 was utilized to align the short reads to the assembled mitogenome for validation purposes, followed by assembly polishing with Pilon v1.2.4 to enhance the quality and accuracy of the final assembly [9].

To confirm the accuracy of the assembled mitogenome, the raw sequencing reads are first aligned to the mitochondrial reference genome using BWA to generate a SAM alignment file. This SAM file is then converted to BAM format and sorted using Samtools v1.21 [10]. Per-position coverage statistics across the mitochondrial genome are calculated from the sorted BAM file using samtools depth and outputted to a text file. This coverage file is imported into R and processed with tidyverse to group the data by position and calculate the mean coverage per position [11]. Finally, the coverage profile across the mitochondrial genome is visualized by plotting the mean coverage per position using ggplot2, allowing inspection of the coverage uniformity across the mitochondrial genome.

The assembled mitogenome of *L. japonica* was then annotated to identify and map the protein-coding genes (PCGs), transfer RNAs (tRNAs), and ribosomal RNAs (rRNAs). Annotation was achieved using a combination of Mitoz v3.4 for PCGs and MITOS for RNA genes [12]. The OGDRAW tool (https://chlorobox.mpimp-golm.mpg.de/OGDraw.html, accessed on 3 April 2023) generated visual maps of the mitogenome, illustrating the gene arrangement and mitogenome organization [13].

### 2.5. Sequence and Phylogenetic Analysis

The base composition and codon usage of the mitogenome were analyzed using CodonW v1.4.4 [14], while patterns of nucleotide diversity and the ratio of non-synonymous to synonymous substitution rates (Ka/Ks) across the PCGs were evaluated with DnaSP v5.10.1 [15]. A sliding window analysis was conducted to examine sequence diversity and identify regions of interest within the PCGs. The window length was set at ≤100 base pairs with a step size of 25 base pairs, allowing for the examination of variations within the PCGs and identification of regions of particular interest. To quantify sequence divergence, pairwise genetic distances were calculated using the Kimura 2-parameter (K2P) model in MEGA v7.0, which has been commonly applied in mitochondrial DNA studies for baseline distance estimates [16]. These distance values were used solely for descriptive comparison of mitochondrial gene variability and not for phylogenetic inference.

Phylogenetic relationships were elucidated using the 13 concatenated PCGs of *L. japonica* and other species from GenBank (Table 1). Sequence alignment was performed using MAFFT v7.471 [17], with ModelFinder determining the optimal phylogenetic model [18]. Maximum likelihood phylogenetic trees were constructed using IQ-TREE v2.1.2, with the GTR + F+R6 model employed, supported by 1000 ultrafast bootstrap replicates [19]. Bayesian inference analyses were conducted with MrBayes v3.2.6, integrating all substitution model parameters, to further validate the phylogenetic relationships [20].

## 3. Results

### 3.1. Genome Characterization and Composition

**Figure 1:**
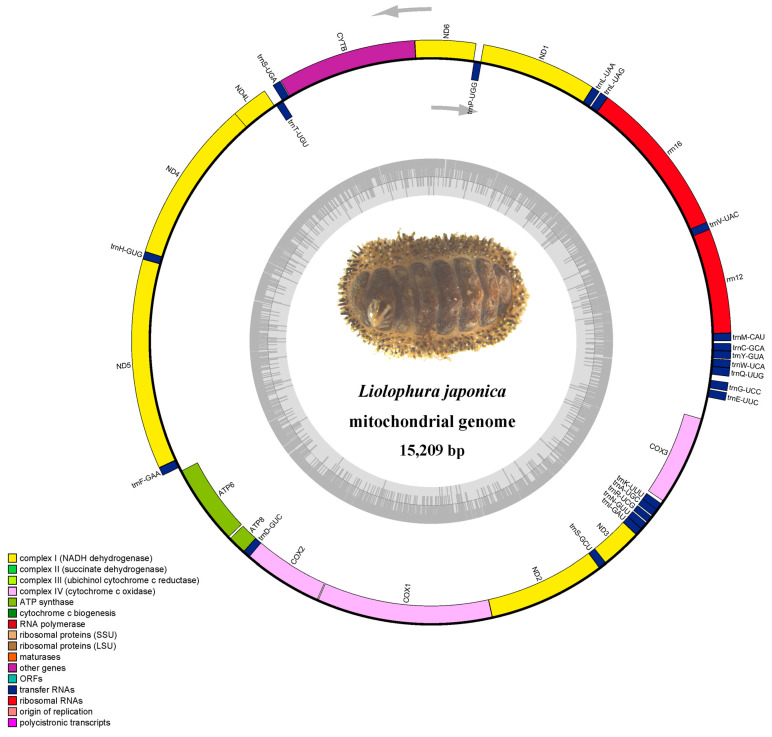
The circular representation of the *L. japonica* mitogenome is depicted, with the outer circle representing the H-strand and the inner circle representing the L-strand. The inner grey circle illustrates the distribution of GC and AT contents, with the dark and light regions denoting the GC and AT contents, respectively.

**Figure 2:**
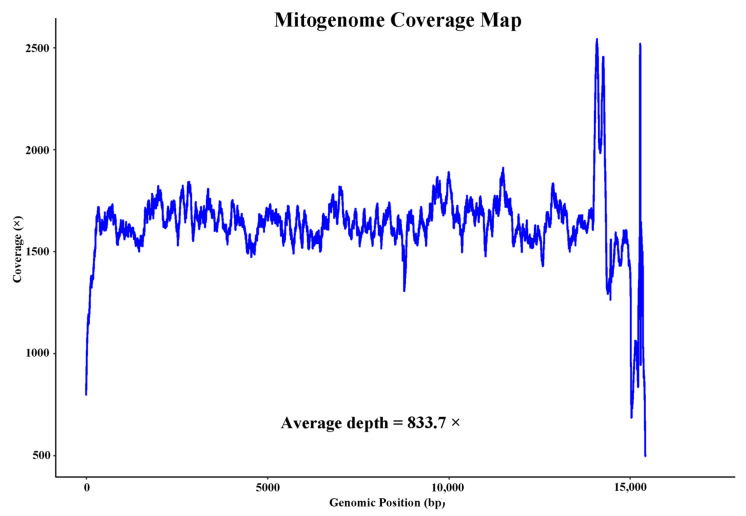
The nucleotide coverage of *L. japonica* mitogenome.

The comprehensive sequencing effort generated 6 gigabases of clean data, enabling the assembly of *L. japonica*’s mitogenome into a circular, double-stranded DNA molecule spanning 15,209 base pairs (Figure 1). The mitogenome was characterized by an average coverage depth of 833.4X, ensuring a high degree of accuracy in the assembly and subsequent analyses (Figure 2). The base composition displayed a slight AT bias—33.14% adenine (A), 13.92% cytosine (C), 16.71% guanine (G), and 36.24% thymine (T)—a trait common among Chitonidae species. This mitogenome encompasses 13 protein-coding genes (PCGs), 22 transfer RNAs (tRNAs), and 2 ribosomal RNAs (rRNAs), and no control region was found in *L. japonica*’s mitogenome (Table 2).

### 3.2. Gene Structure and Variation

**Figure 3:**
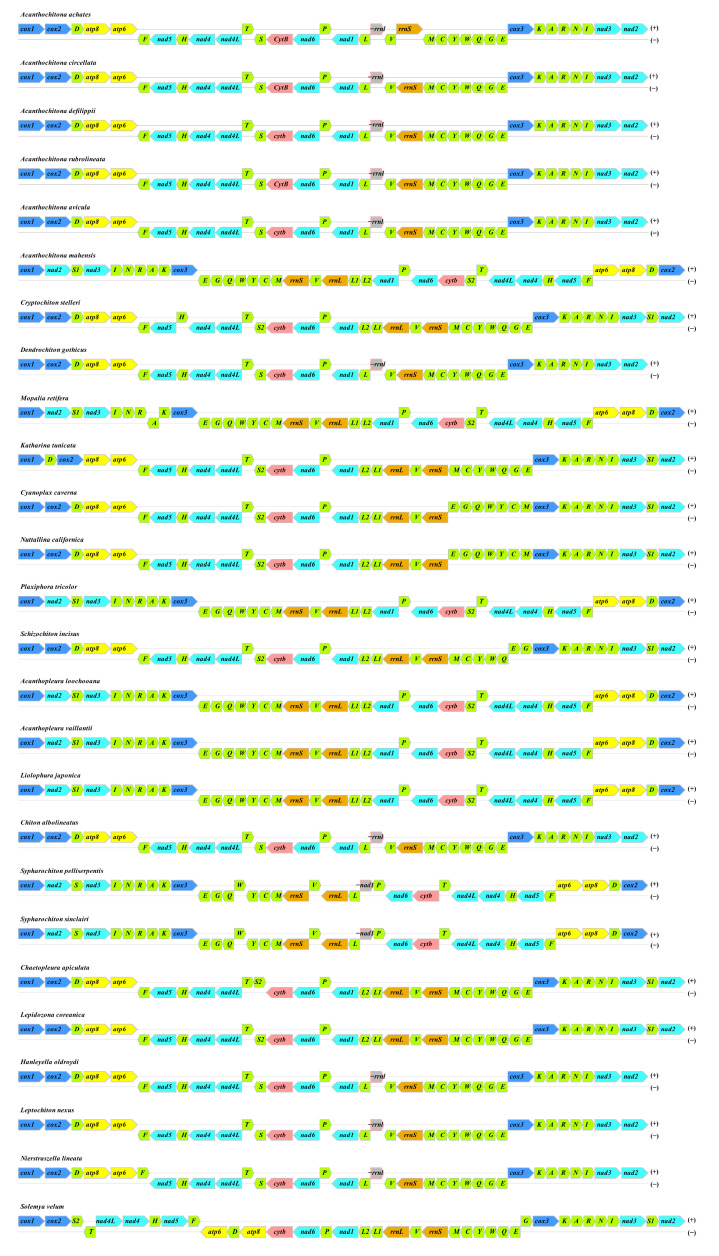
Characters of the 26 mitogenomes in this study. The *L. japonica* in this study was marked in red font.

Transfer RNAs within the mitochondrial genome exhibited variations in length, ranging from 69 base pairs (bp) for tRNA^Arg^ to 74 bp for tRNA^Asn^. In comparison to other mitochondrial genomes within the Chitonidae family, this mitochondrial genome contains a diverse set of genes on the H-strand, featuring vital components such as cob, nad1, nad6, nad4, nad4l, nad5, as well as both the large (l-rRNA) and small (s-rRNA) ribosomal RNA genes. On the other hand, the L-strand is responsible for encoding several crucial genes, including *atp6*, *atp8*, *cox2*, *cox1*, *nad2*, *nad3*, and *cox3* (Figure 3). The distinct gene figuration and composition of *L. japonica*’s mitochondrial genome underscore its structural attributes and point to a sophisticated evolutionary history within the chiton lineage.

### 3.3. Protein-Coding Genes Analysis

Most genes that encode proteins initiate with the ATG start codon. However, *nad*1 and *nad*4 are exceptions, starting with GTG instead. Among these genes, eight terminate with the TAA codon (specifically, *nad*6, *cob*, *nad*5, *atp*8, *cox*2, *cox*1, *nad*3, and *cox*3), while four end with TAG (*nad*1, *nad*4*l*, *atp*6, *and nad*2), and *nad*4 uniquely ends with a T codon. The gene organization and composition identified in this research differ from prior mitochondrial genome studies, suggesting distinct evolutionary pathways due to the preservation of gene structure and sequence.

**Figure 4:**
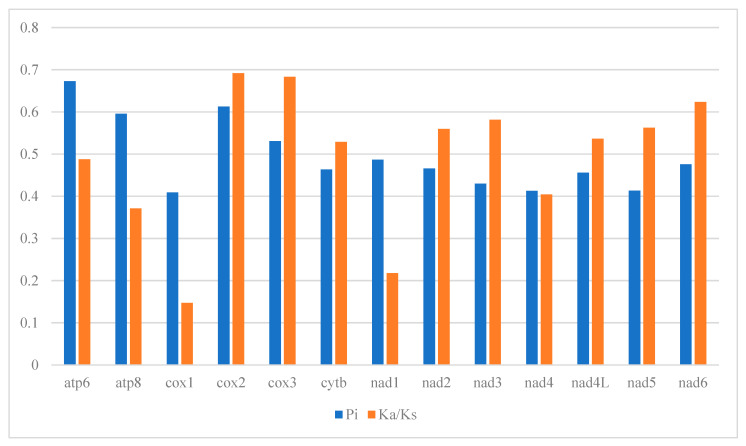
Genetic diversity (Pi) and the Ka/Ks ratio of each PCG among the *L. japonica* mitogenome.

The ratio of nonsynonymous to synonymous substitution rates (Ka/Ks, denoted as ω) serves as a crucial metric in the study of molecular evolution and phylogenetics, facilitating the identification of adaptive changes. In our analysis, all thirteen PCGs presented Ka/Ks values under 1, varying between 0.14 and 0.69 (Figure 4). This pattern underscores the role of purifying selection in the evolutionary development of these genes, highlighting their utility in clarifying phylogenetics. Notably, genes such as *cox*2, *cox*3, *nad*6, and *nad*3 showed slightly elevated Ka/Ks ratios (0.692, 0.683, 0.623, and 0.581, respectively), indicating a comparatively lower evolutionary constraint and the preservation of some non-synonymous mutations. On the other hand, the cox1 gene, with the lowest Ka/Ks ratio at 0.147, experienced the most intense evolutionary scrutiny, underscoring its crucial role in the mitochondrial respiratory chain and inheritance. The stringent purifying selection on cox1 is crucial for mitigating the impact of harmful mutations, affirming its value as a molecular marker for phylogenetic studies. These observations offer valuable perspectives on the evolutionary dynamics and divergence within this group.

Nucleotide diversity, symbolized as π, quantifies the genetic variance between any two nucleotide sequences from a specific gene or genomic segment. This metric sheds light on the genetic diversity present within a gene or the entire genome, enabling researchers to gauge the genetic variation across a population. A higher π value signifies more pronounced nucleotide sequence variation in that area. In our study, we analyzed the nucleotide sequences of 13 PCGs to pinpoint DNA polymorphisms. Among these, the atp6 gene stood out with the highest nucleotide diversity, registering a π value of 0.673, closely followed by cox2 (0.613), atp8 (0.596), and cox3 (0.531). Conversely, the nad4 and cox1 genes had the lowest nucleotide diversity in our dataset, with π values of 0.413 and 0.409, respectively (Supplement Table S1). To delve deeper into the genetic variability, we evaluated the mean genetic distances for these genes. Mirroring the nucleotide diversity findings, genes like nad4l, nad2, nad5, and atp6 exhibited greater genetic distances, marked at 0.78, 0.75, 0.74, and 0.68, respectively. This indicates a broader divergence among sequences within these genes. In contrast, genes such as cox1, cox3, and cob showed lesser genetic distances, at 0.41, 0.46, and 0.46, respectively, pointing to a lower degree of sequence variation.

**Figure 5:**
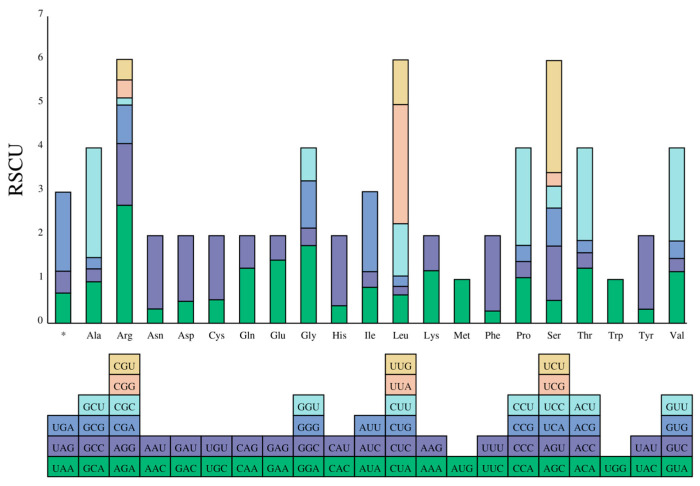
The bar plot shows the distribution of codon content for the amino acids in the 13 protein-coding genes (PCGs) of the *L. japonica* mitogenome. *: represent the stop codon.

The composition of bases, patterns of codon usage, and relative synonymous codon usage (RSCU) are integral to the regulation of gene expression and the stability of mRNA. These aspects also offer key insights into evolutionary trends and the phylogenetic connections among species. Within the mitogenome of *L. japonica*, 13 PCGs are encompassing a total of 3733 codons (Figure 5 and Supplement Table S2). Notably, certain amino acids demonstrate higher frequencies in these codons. Leucine, isoleucine, phenylalanine, serine, valine, and glycine emerge as the most common, contributing to 13.42%, 10.80%, 10.58%, 8.30%, 6.46%, and 6.16% of the total codons, respectively, whereas tryptophan is the least common, making up only 1.04%. These observations underscore the significance of codon usage in unraveling evolutionary trends and deepening our understanding of phylogenetic linkages. The variation in amino acid frequencies across codons may be attributed to multiple factors, such as the specific roles of genes, the evolutionary trajectory of the organism, and environmental influences. Grasping these factors enriches our comprehension of evolutionary dynamics and the interconnectedness of diverse species.

### 3.4. Gene Arrangement

The circular mitochondrial genomes of the 26 polyplacophoran species analyzed here, together with the out-group *S. velum*, each encode the typical complement of 37 genes. Gene order is highly conserved within genera but varies markedly among higher lineages (Figure 3). In Acanthochitona (*A. achates*, *A. circellata*, *A. defilippii*, *A. rubrolineata*, *A. avicula*) and in the chitonids *C. apiculata*, *C. stelleri*, *C. albolineatus*, *L. coreanica* and *S. incisus*, all protein-coding genes are encoded on the heavy strand (H-strand) and follow the ancestral molluscan arrangement *cox1–cox2–trnD–atp8–atp6–trnF–nad5–trnH–nad4–nad4L*.

By contrast, *A. loochooana*, *A. vaillantii*, *L. japonica*, *M. retifera*, *P. tricolor*, and the two Sypharochiton species share a derived architecture in which the block *nad2–trnS1–nad3–trnI–trnN–trnR–trnA–trnK–cox3* is inverted and transferred to the light strand (L-strand). Consequently, when genes are listed clockwise, *cox1* consistently opens the sequence, and *nad2* terminates it.

All genomes preserve the rRNA cassette (trnL_2_-trnL_1_/rrnL-trnV-rrnS) and the downstream *nad1-trnP-nad6-cob* segment, underscoring strong structural constraints on these regions. The out-group *S. velum* retains a more distantly conserved arrangement typical of protobranch bivalves, confirming its suitability for rooting (Figure 3).

These two major patterns, together with minor tRNA translocations (e.g., trnE/G/Q/W/Y/C/M block swapping from H- to L-strand in *Cyanoplax* and *Nuttallina*), map neatly onto the phylogeny (Figure 3) and likely result from tandem-duplication–random-loss events, as proposed for other chitons.

### 3.5. Phylogenetic Analyses

**Figure 6:**
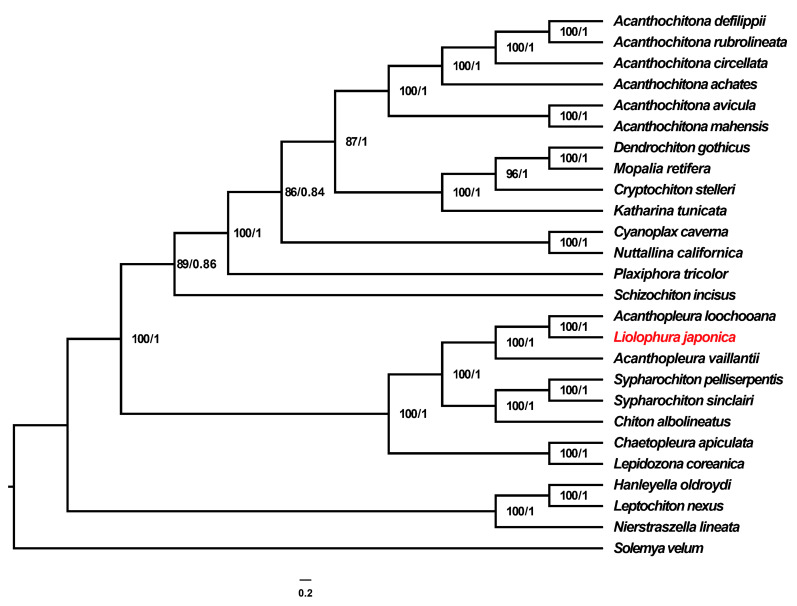
Reconstruction of a phylogenetic tree determined via the maximum likelihood and Bayesian inference methods based on 13 PCGs of 26 mitogenomes. The *L. japonica* in this study was marked in red font.

The mitochondrial phylogeny places *L. japonica* in a well-supported clade with *A. loochooana* and *Acanthopleura vaillantii*, indicating that Liolophura is likely congeneric with Acanthopleura. While we follow current database usage in retaining the name *Liolophura*, our findings reinforce the need for a formal nomenclatural revision of this group (Figure 6). This Chitonidae group is sister to another strongly supported lineage comprising *C. albolineatus*, *Sypharochiton pelliserpentis* and *S. sinclairi*, underscoring distinct intra-familial divergence within Chitonidae. Additionally, *C. apiculata* (family Chaetopleuridae) and *L. coreanica* (family Ischnochitonidae) form a separate yet closely related clade, further highlighting familial relationships within the order Chitonida. Within Chitonida, species of the family Acanthochitonidae split distinctly into two subclades: one comprising *A. achates*, *A. circellata*, *A. defilippii* and *A. rubrolineata* and the other including *A. avicula* and *A. mahensis*. Species of Mopaliidae (*C. stelleri*, *Dendrochiton gothicus*, *M. retifera*, *P. tricolor* and *K. tunicata*) and Tonicellidae (*C. caverna* and *N. californica*) form additional well-supported clades, confirming clear familial boundaries. Representatives of the suborder Lepidopleurida (*H. oldroydi*, *L. nexus* and *N. lineata*) appear basal and phylogenetically distinct from all Chitonida lineages, indicating significant divergence between these two suborders.

## 4. Discussion

The exploration of *Liolophura japonica*’s mitogenome provides profound insights into the evolutionary mechanisms of mitochondrial DNA within the Chitonidae family and extends to the broader molluscan phylum. Notably, the genome exhibits a typical complement of 37 genes but lacks a control region, a feature observed in some other polyplacophoran species. In addition, our findings reveal considerable nucleotide diversity in protein-coding genes such as *atp6*, which may reflect gene-specific selective pressures. These results, while preliminary, highlight the utility of mitogenomic data for exploring evolutionary dynamics and functional divergence within chitons. Further studies with broader taxon sampling are needed to assess the consistency of these patterns across the clade.

The phenomenon of purifying selection observed in *L. japonica*’s PCGs reflects evolutionary pressures to conserve essential metabolic functions. Despite the high mutation rates typically associated with mitochondrial DNA, essential genes, particularly those involved in oxidative phosphorylation, exhibit conservation due to strong functional constraints [21]. This preservation is crucial for maintaining mitochondrial efficacy, especially in energetically demanding environments [22].

Genes such as *cox2* and *nad6* exhibited higher Ka/Ks ratios (0.69 and 0.62, respectively), suggesting relaxed purifying selection compared to *cox1* (Ka/Ks = 0.15). This pattern may reflect functional divergence in energy metabolism pathways or compensatory mutations in nuclear-encoded subunits. For instance, *cox2* encodes a subunit of cytochrome c oxidase, which interacts with nuclear-encoded proteins, potentially buffering mitochondrial mutations. Similar observations in gastropods support the hypothesis that mitochondrial-nuclear coevolution may underlie these dynamics [23,24].

Our mitochondrial phylogenetic analysis offers robust insights into the evolutionary relationships within Polyplacophora, aligning well with recent mitogenomic studies. Significantly, our results precisely position the focal species, *L. japonica*, within a strongly supported Chitonidae clade alongside *A. loochooana* and *A. vaillantii*. This placement corroborates previous morphological and molecular classifications indicating close evolutionary affinities within this family and aligns with the comprehensive mitogenomic framework provided by Irisarri et al. [4]. Our findings align with the 18S rRNA phylogeny, which also places *L. japonica* within a well-supported clade of *Acanthopleura* species [25]. Liolophura belongs to Acanthopleura, and the name of this taxon is Acanthopleura Guilding, 1830. According to the current consensus classification, the class Polyplacophora comprises three extant orders—Lepidopleurida, Callochitonida and Chitonida—with Chitonida further divided into the suborders Acanthochitonina and Chitonina [26]. All taxa sampled in the present study belong to Chitonida, and our mitochondrial tree recovers the subfamily Acanthopleurinae (*Acanthopleura* spp. + *L. japonica*) as a well-supported clade within the family Chitonidae, congruent with recent phylogenomic reconstructions [27]. No topological conflict with the order- or suborder-level framework is observed, reinforcing the robustness of the current classification scheme. While the 18S tree reveals deeper divergences, the mitochondrial data provide higher resolution, particularly for finer relationships within Chitonidae. This congruence between mitochondrial and ribosomal phylogenies enhances the reliability of our results, demonstrating that mitochondrial protein-coding genes are a valuable tool for resolving intra-family relationships within Polyplacophora [28,29].

Notably, this study is limited by the use of a single specimen and restricted taxonomic coverage. Future research should prioritize sequencing multiple individuals from geographically diverse populations and putative cryptic lineages of *L. japonica* to better capture intraspecific genetic variation, population structure, and evolutionary dynamics. Such efforts would provide a more comprehensive understanding of mitogenomic stability, lineage-specific selection pressures, and phylogenetic relationships within the genus. Moreover, integrating additional mitogenomic data from other chiton taxa will be essential for refining intra-familial classifications and testing hypotheses of shared evolutionary history within Chitonidae. These findings provide a genomic foundation for future research on species delimitation, phylogeny, and Polyplacophora.

## 5. Conclusions

Our study successfully delineates the first complete mitochondrial genome in the *Liolophura* genus. The mitochondrial genome of *L. japonica* contains 37 genes essential for mitochondrial function, consistent with the typical gene composition found in other molluscan mitogenomes. The discovery of purifying selection acting on its protein-coding genes underscores the evolutionary pressures to conserve essential metabolic functions, despite the high mutation rates typically associated with mitochondrial DNA.

Phylogenetic analysis based on mitogenomic data placed *L. japonica* in a well-supported clade alongside *A. loochooana* and *A. vaillantii*, suggesting a close evolutionary relationship that may warrant further taxonomic consideration regarding their generic placement. The broader topology recovered major chiton families as distinct clades and confirmed the deep divergence between the suborders Chitonida and Lepidopleurida.

## Figures and Tables

**Table 1:** The reference mitogenome sequence was used to align the sequence in this study.

ID	Superfamily	Family	Organism	Length	AT%
NC_047426.1	Chitonida	Acanthochitonidae	*Acanthochitona avicula* Carpenter	15,203	68.6
NC_082305.1	Chitonida	Acanthochitonidae	*Acanthochitona mahensis* Winckworth	15,013	67.2
NC_087903.1	Chitonida	Acanthochitonidae	*Acanthochitona defilippii* Canefri	14,999	71.7
PQ301026.1	Chitonida	Acanthochitonidae	*Acanthochitona achates* Gould	15,006	68.8
PQ301027.1	Chitonida	Acanthochitonidae	*Acanthochitona circellata* Reeve	14,998	70.3
PQ301028.1	Chitonida	Acanthochitonidae	*Acanthochitona rubrolineata* Lischke	14,986	70.3
KY824658.1	Chitonida	Chaetopleuridae	*Chaetopleura apiculata* Say	15,108	72.8
NC_024173.1	Chitonida	Chitonidae	*Sypharochiton sinclairi* Gray	15,028	72.2
NC_024174.1	Chitonida	Chitonidae	*Sypharochiton pelliserpentis* Quoy & Gaimard	15,048	72.5
NC_047425.1	Chitonida	Chitonidae	*Chiton albolineatus* Broderip & Sowerby I	14,936	72.9
NC_068064.1	Chitonida	Chitonidae	*A. loochooana* Broderip & Sowerby	15,366	70.1
NC_072326.1	Chitonida	Chitonidae	*L. japonica* 1873	15,209	69.3
NC_082877.1	Chitonida	Chitonidae	*Acanthopleura vaillantii* Rochebrune	15,271	67.7
NC_046935.1	Chitonida	Ischnochitonidae	*Lepidozona coreanica* Reeve	16,572	70.2
NC_001636.1	Chitonida	Mopaliidae	*Katharina tunicata* Wood	15,532	69.4
NC_026850.1	Chitonida	Mopaliidae	*Cryptochiton stelleri* Middendorff	15,082	70.5
NC_047424.1	Chitonida	Mopaliidae	*Dendrochiton* gothicus Carpenter	15,288	70.3
NC_068065.1	Chitonida	Mopaliidae	*Mopalia retifera* Thiele	14,984	70.1
NC_085812.1	Chitonida	Mopaliidae	*Plaxiphora tricolor* Thiele	14,909	71
OP994082.1	Chitonida	Schizochitonidae	*Schizochiton incisus* Sowerby	15,615	68.7
NC_026848.1	Chitonida	Tonicellidae	*Cyanoplax caverna* Eernisse	15,141	74.3
NC_026849.1	Chitonida	Tonicellidae	*Nuttallina californica* Reeve	15,604	71
NC_047422.1	Lepidopleurida	Lepidopleuridae	*Leptochiton nexus* Carpenter	15,488	68.9
NC_047423.1	Lepidopleurida	Lepidopleuridae	*Hanleyella oldroydi* Dall	15,692	68
NC_047421.1	Lepidopleurida	Nierstraszellidae	*Nierstraszella lineata* Nierstrasz	15,765	68.9
NC_017612.1	Solemyida	Solemyidae	*Solemya velum* Say	15,660	68.1

**Table 2:** Annotation of genes in the *L. japonica* mitogenome.

Locus	Start	Stop	Size (bp)	Start Coding	Stop Coding	Strand
*tRNA^Met^*	1	66	66			H
12*S rRNA*	66	928	863			H
*tRNA^Val^*	919	985	67			H
16*S rRNA*	967	2338	1372			H
*tRNA^Leu^*	2281	2349	69			H
*tRNA^Leu^*	2362	2427	66			H
*nad* **1**	2427	3378	952	GTG	TAG	H
*tRNA^Pro^*	3370	3435	66			L
*nad* **6**	3438	3936	499	ATG	TAA	H
*cob*	3937	5077	1141	ATG	TAA	H
*tRNA^Ser^*	5082	5150	69			H
*tRNA^Thr^*	5149	5216	68			L
*nad* **4** *l*	5230	5533	304	ATG	TAG	H
*nad* **4**	5526	6879	1354	GTG	T	H
*tRNA^His^*	6874	6937	64			H
*nad* **5**	6937	8653	1717	ATG	TAA	H
*tRNA^Phe^*	8655	8723	69			H
*atp* **6**	8759	9458	700	ATG	TAG	L
*atp* **8**	9477	9639	163	ATG	TAA	L
*tRNA^Asp^*	9639	9705	67			L
*cox* **2**	9703	10,396	694	ATG	TAA	L
*cox* **1**	10,404	11,937	1534	ATG	TAA	L
*nad* **2**	11,938	12,958	1021	ATG	TAG	L
*tRNA^Ser^*	12,961	13,029	69			L
*nad* **3**	13,027	13,384	358	ATG	TAA	L
*tRNA^Ile^*	13,385	13,452	68			L
*tRNA^Asn^*	13,452	13,517	66			L
*tRNA^Arg^*	13,528	13,588	61			L
*tRNA^Ala^*	13,599	13,665	67			L
*tRNA^Lys^*	13,661	13,727	67			L
*cox* **3**	13,754	14,534	781	ATG	TAA	L
*tRNA^Glu^*	14,728	14,794	67			H
*tRNA^Gly^*	14,807	14,876	70			H
*tRNA^Gln^*	14,924	14,993	70			H
*tRNA^Trp^*	14,993	15,059	67			H
*tRNA^Tyr^*	15,065	15,129	65			H
*tRNA^Pro^*	15,129	15,191	63			H

## Data Availability

The genome sequence data that support the findings of this study are openly available in GenBank of NCBI at (https://www.ncbi.nlm.nih.gov/nuccore/NC_072326.1/. accessed on 3 April 2023) under accession no NC_072326. The associated BioProject, SRA, and Bio-Sample numbers are PRJNA916926, SAMN32599642, SRR22986103, respectively.

## Supplementary Materials

The following supporting information can be downloaded at: https://www.mdpi.com/article/10.3390/genes16050606/s1, Table S1: Amino acid composition and relative synonymous codon usage in the mitogenomes of *L. japonica*; Table S2: Nucleotide diversity, Ka, and Ks results; Table S3: Genetic distances, based on 13 PCGs.
